# Over a Thousand Years of Evolutionary History of Domestic Geese from Russian Archaeological Sites, Analysed Using Ancient DNA

**DOI:** 10.3390/genes9070367

**Published:** 2018-07-20

**Authors:** Johanna Honka, Matti T. Heino, Laura Kvist, Igor V. Askeyev, Dilyara N. Shaymuratova, Oleg V. Askeyev, Arthur O. Askeyev, Marja E. Heikkinen, Jeremy B. Searle, Jouni Aspi

**Affiliations:** 1 Ecology and Genetics Research Unit, University of Oulu, 90014 Oulu, Finland; matti.heino@oulu.fi (M.T.H.); laura.kvist@oulu.fi (L.K.); marja.e.heikkinen@oulu.fi (M.E.H.); jouni.aspi@oulu.fi (J.A.); 2 The Institute of Problems in Ecology and Mineral Wealth, Tatarstan Academy of Sciences, 420087 Kazan, Russia; archaeozoologist@yandex.ru (I.V.A.); galimovad@gmail.com (D.N.S.); parus.cyanus@rambler.ru (O.V.A.); art.regulus@mail.ru (A.O.A.); 3 Department of Ecology and Evolutionary Biology, Cornell University, Ithaca, NY 14853, USA; jeremy.searle@cornell.edu

**Keywords:** greylag goose, *Anser anser*, mitochondrial DNA, control region, D-loop, domestication, Medieval Period

## Abstract

The European domestic goose is a widely farmed species known to have descended from the wild greylag goose (*Anser anser*). However, the evolutionary history of this domesticate is still poorly known. Ancient DNA studies have been useful for many species, but there has been little such work on geese. We have studied temporal genetic variation among domestic goose specimens excavated from Russian archaeological sites (4th–18th centuries) using a 204 base pair fragment of the mitochondrial control region. Specimens fell into three different genetic clades: the domestic D-haplogroup, the F-haplogroup that includes both wild and domestic geese, and a clade comprising another species, the taiga bean goose. Most of the subfossil geese carried typical domestic D-haplotypes. The domestication status of the geese carrying F-haplotypes is less certain, as the haplotypes identified were not present among modern domestic geese and could represent wild geese (misclassified as domestics), introgression from wild geese, or local domestication events. The bones of taiga bean goose were most probably misidentified as domestic goose but the domestication of bean goose or hybridization with domestic goose is also possible. Samples from the 4th to 10th century were clearly differentiated from the later time periods due to a haplotype that was found only in this early period, but otherwise no temporal or geographical variation in haplotype frequencies was apparent.

## 1. Introduction

The European domestic goose (*Anser anser*) is one of the few domesticated animals whose evolutionary and domestication history is still largely unknown. As a “minor” domesticate, it is rarely mentioned or discussed in historical documents [1]. Although undoubtedly not as economically or numerically important as the domestic chicken, the domestic goose is quite widely farmed to provide a source of meat, liver (foie gras), eggs, feathers, and down. Historical records indicate that the use of geese, e.g., the fattening of goose for the table and force-feeding, has been known since Egyptian times [2]. The Romans utilized geese extensively for their eggs and meat and also practiced force-feeding to enlarge the livers [1,2]. The feathers of geese were plucked by Romans to be used in cushions and upholstery and quills were utilized for writing since the 5th century CE (CE: Common Era; BCE: Before Common Era) [2]. Geese have also been used as guards due to their loud cackling [2]. In addition to their economical use, geese have had a religious significance in certain cultures, e.g., Roman Egypt, Asia Minor, Greece and Roman Italy [1].

The European domestic goose is descended from wild greylag goose (*A. anser*) [3,4] and based on its pink bill coloration more likely from the eastern subspecies (*Anser anser rubrirostris*) than the nominate western subspecies (*Anser anser anser*) [5]. The domestication process of the greylag goose followed the prey pathway, in which the species was first being hunted before more intensive herd-management started [6]. The goose is easy to domesticate from goslings and has a natural tendency to gather fat for migration, which has been exploited to make the wild goose too heavy to fly [2]. Geese have also been domesticated in southeast Asia but derived from another species, the swan goose (*Anser cygnoid*), and the domesticate is known as the Chinese goose [3,7,8,9,10]. The European domestic goose and Chinese domestic goose can readily hybridize with each other [7,11], and the European domestic goose is known also to hybridize with its wild counterpart [2,11]. Hereon, use of the term “domestic geese” relates to European domestic geese unless otherwise stated.

It has been proposed that geese were domesticated around 3000 BCE in southeastern Europe [7], possibly in Greece [2] (for a review see [1]), but reliable evidence of domestic geese comes from a much later period (8th century BCE) in *The Odyssey* [2]. Another potential domestication site is in Egypt during the Old Kingdom (2686–1991 BCE) due to iconographic evidence of goose exploitation, but this scenario for the original domestication event has been considered less likely [2]. Geese were also herded by ancient Mesopotamians for food and sacrifices and depicted in Mesopotamian art from the early Dynastic Period (2900–2350 BCE) onwards [2]. Certainly, fully domesticated geese were present during the New Kingdom times in Egypt (1552–1151 BCE) and contemporaneously in Europe [2], and goose husbandry involving several varieties was well established by the Romans by the 1st century BCE [1]. In the Medieval Period, goose husbandry was at its peak with large flocks kept by peasants [1]. Archaeological evidence of the domestic goose in northern Europe indicates that it was probably introduced into Scandinavia during the Early Iron Age (400 BCE–550 CE), and domesticated geese definitely appeared there by the Late Iron Age onwards (550–1060 CE) [12]. 

Mitochondrial DNA (mtDNA) analysis of modern geese has been employed to make inferences about domestication and has demonstrated that modern domestic geese were derived from a limited genetic base [11]. However, it was not possible to interpret if the observed low diversity dated back to the time of domestication or if it was of a more recent origin [11], perhaps originating with the creation of the modern breeds some hundreds of years ago. Analysis of archaeological and museum samples from different time periods, but of the same geographical area, may make it possible to parse out the genetic signature of domestication and the formation of modern breeds [13].

Goose bones found from archaeological contexts often lack suitable morphological criteria to distinguish domestic individuals from their similarly sized wild forms [1] and usually the classification of goose bones to domestic and wild forms is not even attempted. Ancient DNA (aDNA) studies could overcome the limitation of identification of wild and domestic goose bones in archaeological contexts but there have been very few studies on aDNA in geese. In a recent aDNA and domestication review, goose was not even mentioned [13] although there was a small-scale study by Barnes et al. [14] to separate wild and domestic geese at archaeological sites in the UK. Ancient DNA studies on other goose species have also been scarce and focused mainly on species identification [15,16,17] or for studying genetic diversity [18]. 

A large collection of domestic goose bones became available from 15 archaeological sites in Russia, providing an unprecedented opportunity to apply aDNA analysis to domestic geese over a wide timescale. Unlike many previous studies, comparative skeletal collections were used to classify goose bones as wild or domesticated (see [19]). Earlier published sources [20,21,22] were also followed to determine criteria for the separation of domestic and greylag goose bones. The samples apparently cover the whole history of domestic geese in the Middle Volga Region spanning from the onset of Medieval Period (4th–5th centuries) to the 18th century (Table S1). None of the previous goose aDNA studies have used such a wide temporal scale or studied domestic goose in such a northern location. These aspects are of interest but also aDNA time-series like this can potentially provide insights into the overall domestication history of the domestic goose. 

In our study, we not only analyzed the temporal genetic variability among the Russian domestic geese from different time-periods, but we also compared the genetic diversity between the ancient samples and modern domestic goose breeds. We used a part of the mtDNA control region as our genetic marker because the high substitution rate in this non-coding region makes it very valuable in a species which otherwise has low sequence variability [23]. Also, mtDNA preservation is better than for nuclear DNA in ancient samples because of the higher copy numbers in cells. In addition, a large reference database of modern samples exists for mtDNA. To gain greater knowledge on the origin and evolution of the European domestic goose, the aims of our study were (1) to establish if modern domestic goose haplotypes were present in the Russian archaeological samples, (2) to determine if there is temporal change in haplotype proportions in samples from different historical periods, and (3) to estimate temporal fluctuations in genetic diversity in those periods.

## 2. Materials and Methods

### 2.1. Sample Material

**Figure 1:**
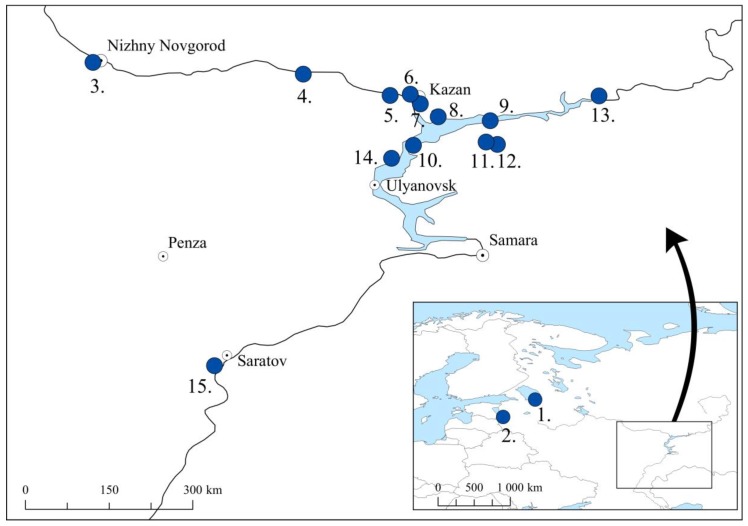
Archaeological locations for subfossil domestic geese from 4th–18th century CE (Common Era): 1. Staraya Ladoga (Leningrad Region), 2. Pskov city (Pskov Region), 3. Nizhny Novgorod Kremlin (Nizhny Novgorod Region), 4. Chebosakry city (Chuvash Republic), 5. Sviyazhsk (Tatarstan Republic), 6. Kazan Kremlin (Tatarstan Republic), 7. Kazan State University, Kazan city (Tatarstan Republic), 8. Imenkov hillfort (Tatarstan Republic), 9. Ostolopovskoe settlement (Tatarstan Republic), 10. Bulgar (Tatarstan Republic), 11. Toretskoe settlement (Tatarstan Republic), 12. Bilyarsk (Tatarstan Republic), 13. Elabuga hillfort (Tatarstan Republic), 14. Tetyushkoe II hillfort (Tatarstan Republic), and 15. Bagaevskoe settlement (Saratov Region).

Subfossil goose bones (*n* = 67) classified as domestic geese were derived from 4th–18th century CE Russian archaeological sites in Tatarstan Republic (*n* = 51), Saratov Region (*n* = 1), Chuvash Republic (*n* = 4), Nizhny Novgorod Region (*n* = 3), Leningrad Region (*n* = 5), and Pskov Region (*n* = 3) (Figure 1, Table S1). The archaeological context and identification of bird remains from the Volga Region (Tatarstan, Saratov, Chuvash, and Nizhny Novgorod) were previously described in [19,24], and the site descriptions from the Leningrad and Pskov Regions in [25].

### 2.2. DNA Extraction and Amplification

For each goose bone fragment sampled, the outer layer of the bone was removed using a drill (Dremel, Breda, The Netherlands), and subsequently about 50–150 mg of bone powder was collected, depending on the size of the fragment. DNA extractions were performed using a silica spin column–based protocol originally published by Yang et al. [26] as modified in [27,28] with slight modifications. Samples were first pre-lysed in 1 mL of lysis buffer (EDTA 0.45 M, *N*-layrylsarcosyl 0.5% and proteinase K 0.25 mg/mL), incubating samples for 50 min at 55 °C under rotation. The lysis solution was removed by centrifuging for 2 min at 11,300 *g*, and the supernatant was discarded. Then, 1 mL of fresh lysis buffer was added to the pelleted bone powder and samples were incubated for 1 h at 55 °C and then overnight at 37 °C under rotation. The second lysis fraction was centrifuged for 2 min at 12,000 *g* and used for DNA extraction as the second fraction should be enriched in endogenous DNA [28,29,30,31,32].

The supernatant from the second lysis fraction was mixed with 3 mL of 10 mM Tris-EDTA buffer and concentrated with Amicon^®^ Ultra-4 Centrifugal Filter Unit 30 kDA (Merck Millipore, Darmstadt, Germany) to 250 μL by centrifuging for 20 min at 1000 *g*. The flow-through was discarded, 3 mL of 10 mM Tris-EDTA buffer was added, and samples were concentrated to a final volume of 100 μL by centrifuging for 20–30 min at 1000 *g*. DNA was extracted from the concentrate using MinElute PCR purification Kit (Qiagen, Hilden, Germany) according to manufacturer’s instructions using the centrifugal speeds from [28] with the exception of eluting the DNA to 50 μL of preheated (60 °C) EB buffer with 0.05% final concentration of TWEEN^®^ 20 (Sigma-Aldrich, Saint Louis, Missouri, USA). The column was left to stand for 5 min, centrifuged at 12,000 *g* and this step was repeated, yielding 100 μL of eluted DNA that was stored in −20 °C.

**Figure 2:**
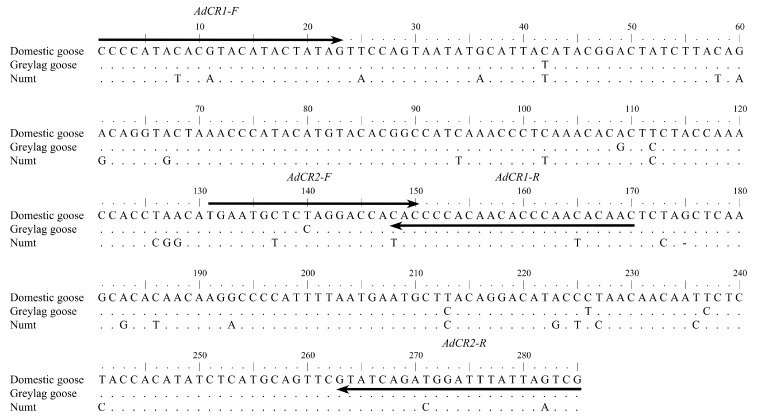
Alignment of domestic goose (GenBank accession number: GQ120441), greylag goose (AF159961) and Numt (nuclear sequence of mitochondrial origin; AF159970) sequences of the hypervariable part of the mitochondrial control region domain I (285 bp). The designed primer pairs AdCR1 and AdCR2 are shown as black arrows. The primer sequences were removed, and the remaining two fragments were concatenated for later analysis.

We designed primers that amplify the hypervariable portion of the mitochondrial control region domain I for the geese studied and which contain mismatches to Numts (nuclear sequences of mitochondrial origin [33], for review see [34]). Precautions to avoid amplification of Numts in geese have been previously published in [23]. Because of the Numt involving the control region, we were not able to obtain primer pairs that would amplify overlapping fragments as would normally be preferred. Instead, we used primer pairs AdCR1-F/AdCR1-R (AdCR1-F: 5′CCCCATACACGTACATACTATAG, AdCR1-R: 5′GTTGGGTGTTGTGGGGTG) and AdCR2-F/AdCR2-R (AdCR2-F: 5′TGAATGCTCTAGGACCACAC, AdCR2-R: 5′CGACTAATAAATCCATCTGATAC) that overlap only by their primer sequence (Figure 2). After the removal of the primer sequences, the sequences consisted of two fragments that could be subsequently concatenated. The primer pair AdCR1-F/AdCR1-R amplified 123 base pairs (bp) and the primer pair AdCR2-F/AdCR2-R amplified 111 bp resulting in a 204 bp concatenated sequence. The haplotype sequences are available in GenBank with accession numbers MH491822-MH491827. We performed PCR in 25 μL reaction volumes using 1× PCR buffer (HotStarTaq, Qiagen), 0.2 μM of each primer, 0.2 mM dNTPs, 2.5 mM MgCl_2_, 1 mg/mL BSA (Bovine serum albumin), KAPA Taq HotStart polymerase (Kapa Biosystems, Wilmington, Massachusetts, USA) and 2 μL of extracted DNA. The thermal profile consisted of 95 °C for 10 min, followed by 55 cycles of 94 °C for 30 s, 57 °C for 30 s and 72 °C for 30 s with a final extension of 72 °C for 7 min. Each DNA fragment was amplified at least twice. We used BigDye Terminator v.3.1 (Applied Biosystems, Foster City, California, USA) for sequencing with the PCR-primers and the reactions were run on an ABI 3730 (Applied Biosystems). Sequences were manually edited using the program CodonCode Aligner v.4.0.4. (CodonCode Corporation,, Centerville, Massachusetts, USA) and aligned using the ClustalW algorithm [35] implemented in the CodonCode Aligner.

### 2.3. Authentication

Several measures were followed to avoid contamination of the ancient samples [36]. The DNA extractions and the pre-PCR work were carried out at the Center of Microscopy and Nanotechnology at the University of Oulu, Finland in dedicated aDNA clean room facilities that were physically separated from the modern DNA and the post-PCR facilities. Further, the bone drilling was performed within a separate room from the DNA extraction and PCR preparation. The PCR-reaction setup was performed in a separate dedicated UV sterilizing PCR workstation (Peqlab, Fareham, United Kingdom). Negative controls were used in the extractions and the PCRs and the amplifications were performed at least twice for each sample to identify post-mortem base modifications. For final analyses, we only accepted sequences that were sequenced successfully at least twice from both strands. In addition, amplification of longer fragment (170 bp including the primers) showed a large decrease in amplification success, in line with the fragmentary nature of ancient DNA.

### 2.4. Sequence Analyses

**Figure 3:**
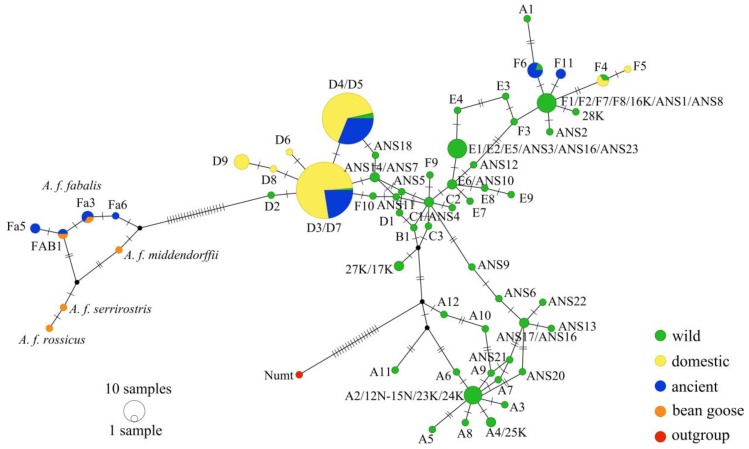
Median-joining haplotype network of the concatenated hypervariable part of the mitochondrial control region (204 bp) of subfossil goose samples (ancient), modern domestic goose samples (domestic), modern wild greylag goose *Anser anser* haplotypes (wild), bean goose *Anser fabalis* haplotypes (bean goose), and nuclear sequence of mitochondrial origin, Numt (outgroup) sequences. Forward slashes between haplotype names denote haplotypes that differ over the whole control region sequence (1249 bp) but cannot be distinguished in the 204 bp sequence analyzed here. Circle area is proportional to the frequency of each haplotype and mutational steps between the haplotypes are indicated by tick marks across branches.

We used the program BioEdit 7.2.5 [37] to align our sequences with those obtained from GenBank and the modern sequences from [11]. The Heikkinen et al. [11] sequences included greylag goose haplotypes classified into six different clades (A–F) and individual domestic goose sequences (*n* = 101) from the clades D and F. Haplogroup D consists of majority of known domestic goose haplotypes (D3–D9) while the rest of the domestic goose haplotypes are found within the F-haplogroup (F4–F5) along with wild greylag goose haplotypes (Figure 3). The domestic haplogroup D contains also wild greylag geese that have most probably a recent hybrid origin between wild and domestic geese [11]. A variety of domestic goose breeds are listed in Heikkinen et al. [11]. We obtained GenBank sequences of wild greylags (AF159961–AF159963 [23], KT276333–KT276355 [38] and EU601724–EU601734 [39]), domestic geese (GQ120441 [4]) and bean geese (*Anser fabalis*; EU186807, EU186812, EU186810, EU186805 [40] and MH491808 [41]). We constructed a median-joining network [42] implemented in the program PopART [43] using an ε value of zero. Following Bensasson et al. [44] and Heikkinen et al. [11], we used the Numt sequence AF159970 [23] as an outgroup for the network. We also constructed a temporal statistical parsimony network for the ancient samples, the modern haplotype groups that contain the domestic geese (D- and F-haplogroups), and the modern bean goose sequences using the TempNet [45] R-script [46].

We estimated the genetic diversity of the domestic geese for different historical time periods. We calculated the number of haplotypes (*H*), haplotype (*h*) and nucleotide (*π*) diversities, Tajima’s *D* and Fu’s *Fs* in each temporal group using DnaSP v.5 [47]. We tested if the diversity estimates *h* and *π* differed between these historical time periods and ‘the Present Period’ using one tailed *t*-tests (Welch’s *t*-test). The presence of genetic structure among the temporal samples was investigated using analysis of molecular variance (AMOVA [48]) as implemented in Arlequin 3.5.1.3 [49]. We used this software also to estimate genetic distance among the three temporal groups with pairwise *Φ*_ST_ using the Kimura 2-parameter genetic distance [50] and tested for significance with 10,000 permutations. We chose the Kimura 2-parameter substitution model according to AIC (Akaike Information Criteria; 1279) and BIC (Bayesian Information Criterion; 3570) values in the MEGA7 program [51]. The best-supported substitution model was the HKY [52], but this model is not implemented in the Arlequin program, so the second best-supported model was used. We applied a sequential Bonferroni correction [53] to the *Φ*-statistics.

## 3. Results

### 3.1. Mitochondrial DNA Haplotypes

In total, we obtained sequences from 46 of the 67 archaeological bones sampled. The overall sequencing success rate was 76%. The sequencing failed especially for the oldest (4th–8th century) samples with the success rate of only 36% for these samples. Twenty-one individuals were excluded as follows: PCR amplification failed completely for eight samples, was only sporadically successful for another eight samples, and five samples were possible duplicates of already sequenced individuals.

Eight haplotypes were found in our study, of which three (F11, Fa5, and Fa6) were new, and the remaining five haplotypes were previously described. The new haplotypes can be attributed to previously described lineages, and we used the existing nomenclature [11,41] to name the haplotypes. We found 30 variable sites among the ancient geese (Table S2). The haplotypes found in our study belonged to three different lineages shown in Figure 3: haplogroup D, haplogroup F, and the taiga bean goose (*Anser fabalis fabalis*). Haplogroup D contains the majority of known domestic goose haplotypes while the F-haplogroup harbors the rest of the known domestic goose haplotypes along with wild greylag goose haplotypes (see Section 2 above). These haplogroups were well-separated in the haplotype network (Figure 3), although some caution in interpretation is necessary. This network is based on a short control region sequence (204 bp) and has misplaced some haplotypes classified on the basis of the whole control region (1249 bp [11]), most notably A1 and F10. Sequences classified as D4 and D5 based on 1249 bp could not be separated on the basis of the 204 bp sequence that we studied, and so are designated D4/D5; likewise, for sequences D3 and D7.

**Figure 4:**
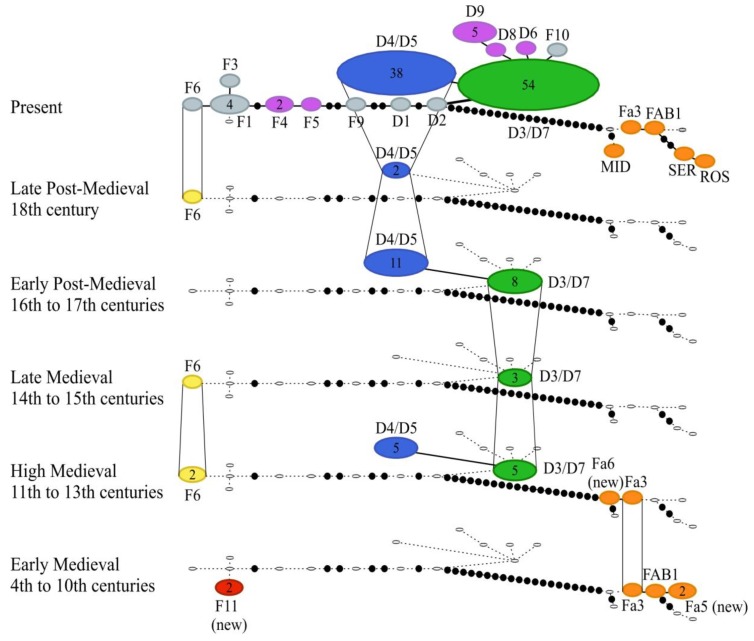
Temporal statistical parsimony network of the concatenated hypervariable part of the mitochondrial control region (204 bp) of subfossil goose samples from Russia from the 4th to 18th century CE and modern greylag goose (*Anser anser*) haplogroups D and F. These groups contain the known domestic goose haplotypes D3 and D7 (green), D4–D5 (blue), and D6, D8–D9, and F4–F5 (purple), and the wild greylag goose in grey color in the depiction for the Present Period. Selected modern bean goose (*Anser fabalis*) haplotypes are also shown separated by at least 21 mutational steps from the others (orange color). MID, SER, and ROS denote the subspecies *Anser fabalis middendorffii*, *Anser fabalis serrirostris*, and *Anser fabalis rossicus,* respectively, while FAB and Fa denote *Anser fabalis fabalis* haplotypes. The size of each ellipse is proportional to the frequency of each haplotype and the number of individuals greater than one are indicated with a number within the ellipse. Small white ellipses indicate haplotypes absent in that time period. Number of black dots +1 connecting haplotypes equals to nucleotide differences.

Sixteen of the ancient geese carried the D3/D7 haplotype, while 18 individuals carried the D4/D5 haplotype (Figure 3, Table S1), representing domestic geese. Both the D3/D7 and D4/D5 haplotypes appeared during the High Medieval Period from the 11th century onward, and their presence continued until the present day (Figure 4). These haplotypes were about equally common in the ancient samples except in the Late Medieval Period (14th to 15th century) the D4/D5 haplotype was absent and in the Late Post-Medieval Period (18th century) the D3/D7 haplotype was absent. However, the samples sizes were low for these periods. During the Present Period D3/D7 (*n* = 38) and D4/D5 (*n* = 54) are the most common haplotypes among the domestic geese. In addition, a few wild greylag geese harbor D3/D7 and D4/D5 haplotypes in the Present Period (Figure 3). Twenty-five of the ancient D-haplogroup samples were attributed to domestic goose based on bone morphology, but for the remainder, there was uncertainty whether the bones were from domestic or wild birds (Table S1).

Six individuals carried haplotypes belonging to the F-haplogroup (Figure 3), with four individuals having haplotype F6 that is associated with contemporary individuals of the wild greylag goose from Lake Kulykol, Kazakhstan [11]. The remaining two individuals possessed a previously undescribed haplotype F11 that was only present in the Early Medieval temporal group (4th to 10th century; Figure 4). Based on the morphological identification, the bones containing the F11 haplotype were assigned to either domestic or domestic/wild greylag (Table S1). Thus, whether this haplotype represents wild greylag or true domestic goose is uncertain. The haplotype F6 appeared in the fossil record during the High Medieval Period (11th to 13th century) and was present in all of the time periods except for Early Post-Medieval Period (16th to 17th century). Half of the subfossil bones carrying F6 haplotypes were identified as domestic and half as domestic or wild greylag goose based on morphology (Table S1).

Six samples were of a different species—the taiga bean goose (*Anser fabalis fabalis*). One of these had an FAB1 haplotype that has been found in Norway, Sweden, Finland, western Russia, and western Siberia (Table S1 in [40]). Two had a haplotype Fa3 that has been identified as the most common haplotype among the taiga bean geese hunted in Finland [41]. Two other previously undescribed taiga bean goose haplotypes Fa5 and Fa6 were also detected, these were closely related to haplotypes FAB1 and Fa3 (Figure 3). The taiga bean geese were restricted to the two earliest time periods: the Early Medieval (4th to 10th century) and the High Medieval (11th to 13th century; Figure 4). Almost all of the taiga bean goose bones had been identified as domestic geese based on bone morphology, only two bones had been considered doubtful, representing either domestic or wild greylag goose (Table S1) but not the bean goose.

**Figure 5:**
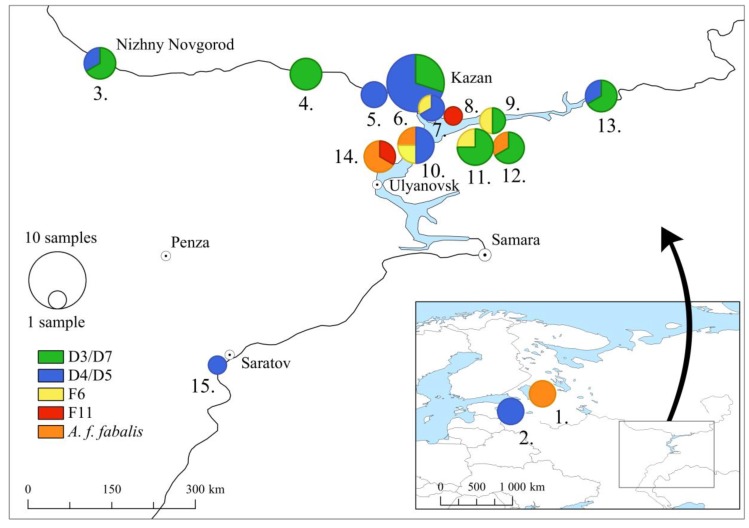
Haplotype frequencies in ancient goose samples from archaeological sites based on the concatenated hypervariable part of the mitochondrial control region (204 bp). The sampling locations are as follows: 1. Staraya Ladoga (Leningrad Region), 2. Pskov city (Pskov Region), 3. Nizhny Novgorod Kremlin (Nizhny Novgorod Region), 4. Chebosakry city (Chuvash Republic). 5. Sviyazhsk (Tatarstan Republic), 6. Kazan Kremlin (Tatarstan Republic), 7. Kazan State University, Kazan city (Tatarstan Republic), 8. Imenkov hillfort (Tatarstan Republic), 9. Ostolopovskoe settlement (Tatarstan Republic), 10. Bulgar (Tatarstan Republic), 11. Toretskoe settlement (Tatarstan Republic), 12. Bilyarsk (Tatarstan Republic), 13. Elabuga hillfort (Tatarstan Republic), 14. Tetyushkoe II hillfort (Tatarstan Republic), and 15. Bagaevskoe settlement (Saratov Region).

Geographically, the haplotype distribution did not show clear trends (Figure 5). In three sampling sites (Nizhny Novgorod, Kazan Kremlin, and Elabuga hillfort), both D3/D7 and D4/D5 haplotypes were present, but in most of the sites, only one of these two main haplotypes were found. Haplotype F6 was present in four geographically close sampling sites: Kazan city, Bulgar and Ostolopovskoe and Toretskoe settlements. Haplotype F11 was restricted to the Imenkov hillfort and the Tetyushkoe II hillfort. The Staraya Ladoga archaeological site contained only taiga bean geese, and three sites (Bulgar, Bilyarsk, and Tetyushkoe II hillfort) in the Middle Volga Region contained taiga bean geese along with the domestic geese.

### 3.2. Genetic Diversity

Genetic diversity of the domestic geese was compared between different time periods. The bean goose haplotypes were excluded from this analysis as exclusively *A. anser* haplotypes are found in modern domestic geese [11], and it is their derivation that was the focus of this study (although the recovery of bean goose haplotypes is of considerable interest and is considered in the Discussion; see below). The groupings compared were the High and Late Medieval Periods (11th–15th centuries CE), the Post-Medieval Period (16th–18th centuries CE) and the Present Period (Table 1). The combining together of the two Medieval Periods and the two Post-Medieval Periods was to increase sample sizes. The Early Medieval Period was not combined with the other Medieval Periods as it would have resulted in an extremely long time period (4th–15th centuries CE), with a heterogeneity that may introduce bias. In addition, the sample size for the Early Medieval Period was small (*n* = 2). The number of haplotypes was the highest in the Present Period, but that is not surprising given the large sample size. The nucleotide and haplotype diversities were the highest in the Medieval Period (*p <* 0.001; Table 1), but given the long time period of samples, this should be viewed with caution. The very high nucleotide diversity in the Medieval Period relates to the relatively high frequency of haplotype F6 individuals (Figure 4). Tajima’s *D* and Fu’s *Fs* were not significant for any of the temporal groups.

We used AMOVA to partition the genetic diversity among the different temporal groups. The among group variation explained 6.2% of the total genetic variation, while the within group variation explained 93.8%. The pairwise *Φ*_ST_ value (0.11, *p* = 0.02) was high and statistically significant between the Present and the High and Late Medieval groups, indicating that these groups are the most differentiated (Table 2).

## 4. Discussion

In this study, we have identified three genetic lineages among Medieval and Post-Medieval domestic goose samples from Russian archaeological sites: the main domestic D-haplogroup, F-haplogroup, and taiga bean goose haplotypes. We used the modern breeds from Heikkinen et al. [11] as representatives of current variation among modern domestic geese. In Russia, the domestic goose breeds have either European origin or the local geese have been crossbred with European or Chinese breeds [54]. Most of our samples were excavated from the Middle Volga Region that is currently dominated by 10 breeds of domestic geese, which all belong to or are in one way or another connected with European breeds of geese [55]. In addition, some breeds are crossed with or have a direct relationship with Chinese domestic goose breeds [55]. Thus, the breeds in Heikkinen et al. [11] should represent well the variation also in contemporary Russia. No Chinese ancestry was detected in our samples, so presumably the crossbreeding of these geese is a more recent phenomenon. However, we cannot detect if the European geese have been mated with Chinese goose ganders with maternally inherited mtDNA.

The most common mitochondrial haplotypes among the modern domestic goose D3 and D4 (D3: *n* = 53 and D4: *n* = 33 [4,11]) were also the most common haplotypes in these Russian archaeological sites. However, the sequenced fragment of the mtDNA control region could not differentiate between haplotypes D3 and D7 or D4 and D5, thus haplotypes D5 and D7 might actually be present in the ancient sample. In any case, the haplotypes D3/D7 and D4/D5 appear to be domestic haplotypes, as among modern specimens, they are restricted to domestic geese, except for a few wild individuals in Scotland (haplotype D4), The Netherlands (haplotype D3) [11], and Norway (haplotype ANS19 in [38], which is a partial sequence of D5 in Heikkinen et al. [11] and also in Wang et al. [4]), which probably have a hybrid (wild × domestic) origin. This presence of apparently “domestic” haplotypes in the archaeological record is of interest for domestication history. During the domestication process, the first genetic bottleneck occurs during the early phase of domestication when a subset of a population is selected for domestication [56,57]. Illustrations from the Old Kingdom Egypt show already diverse coloration in geese and during Roman times, several goose varieties were recognized, such as mottled and white types [1]. The breeds Embden, Toulouse, Sebastopol, and the swan goose derivatives “Chinese” and “African” were known prior to the mid-19th century [5,7,58] and could have served as a basis for modern breeds. The second bottleneck occurs with origin of modern breeds when certain desirable traits are selected for [56,57]. As our samples pre-date the modern breeds, which were mostly formed due to intensive selective breeding within the past 200 years [59,60], it is possible that the dominance of D3/D7 and D4/D5 is due to the first domestication bottleneck. We did not detect other domestic haplotypes (D6, D8, D9, F4 or F5) found only in domestics in the survey of Heikkinen et al. [11] suggesting that these haplotypes could be of a more recent origin. This could imply that the modern breeds are derived from a limited gene pool that possibly traces back to the original domestication event.

Considering the F-haplogroup, there could be a variety of explanations for its presence in the ancient samples. In modern samples this haplogroup was found in non-breed Turkish domestic geese, wild greylag geese from Iran and Kazakhstan and wild greylag geese from The Netherlands and Denmark that are most probably descendants of introduced eastern *Anser anser rubrirostris* geese [11]. First, all ancient samples belonging to the F-haplogroup could in fact be hunted wild greylag geese that were misidentified as domestic geese based on bone morphology. Second, individuals belonging to the F-haplogroup could be the descendants of hybridization between the domestic and wild geese. In this case, a wild goose of the F-haplogroup must have mated with a domestic gander as mtDNA is maternally inherited. However, this mating between adult geese seems unlikely as geese tend to mate for life and females are philopatric to their natal sites [61]. It is also possible that wild goslings or eggs were collected and raised, providing opportunities for hybridization. Goslings can readily imprint to humans, a feature that has substantially helped in taming wild geese. In Eurasia, it has been common to collect eggs and goslings and further raise them in captivity partly, for companionship and partly as a source of food [62,63,64,65,66,67,68,69,70,71,72,73] (see Text S1). Incorporation of wild forms into the domestic gene pool has been practiced with several other domestic species as well (pig [74,75], cattle [76], horse [77,78], donkey [79], and dromedary [80]). Third, the F-haplotypes could represent an independent domestication event of the local eastern greylag geese, as this haplogroup seems to be more typical for the eastern *Anser anser rubrirostris* geese [11]. This would imply that the goose was domesticated at least twice; however, this scenario does not have the support of any other evidence pointing to several domestication events.

Considering the individual haplotypes within the F-haplogroup, the F11 haplotype was only found from the Early Medieval Period (4th to 8th century) in the Middle Volga Region. These samples have been associated with an ethnic group called the Imenkov culture. This culture had a distinctive ethnic composition, economic activity, and, in particular, the use of domestic animals, which distinguished them from subsequent cultures, which were an ethnic combination of Turkic and Finno-Ugric [81]. This change in the ethnic composition in the Middle Volga Region could explain why the goose haplotypes from the Early Medieval Period differed from the later time periods. These bones are from immature or subadult birds, suggesting they were domestic rather than wild [19] and thus possibly representing an extinct lineage of domestic geese. This would imply that the first domesticated geese appeared in the Middle Volga Region with the onset of the Medieval Period (4th–10th centuries). However, it cannot be ruled out that these individuals were goslings collected from the wild. Overall, the first morphologically identified domestic geese appeared in the archaeological record in the European part of Russia and Ukraine in 500 BCE–300 CE and several settlements harbored domestic geese during the Medieval times [19,21,24,82,83,84,85,86,87,88,89] (see Text S1). In western Siberia, the domestic goose appeared much later (16th–17th century) [90,91].

Haplotype F6 was present in all temporal groups except in the Early Medieval Period (4th to 10th century) and the Early Post-Medieval Period (16th to 17th century). From the Present Period, it has only been found from one modern wild greylag goose from Kazakhstan [11]. This haplotype could have originated from wild hunted greylag geese or from a domestic goose lineage that has either gone extinct or has remained undetected in previous studies. It might be possible to find F-haplotypes from some local domestic goose breeds, such as the domestic geese bred by Udmurts and Maris that had several greylag goose characteristics [92] (see Text S1), the Shadrin breed that originates from local wild and domestic geese [51] (see Text S1) or the Javakhetian or Bogdanovski breed from Georgia that has been claimed to have descendent directly from local wild geese [54]. The domestic origin of the subfossil geese carrying haplotype F6 is the most supported alternative, because this haplotype was found in almost all temporal groups from the 11th century onward with no other F-haplotypes. A more varied selection of F-haplotypes would have been expected if the geese would have been hunted individuals. The absence of F6 in the Early Post-Medieval Period could be due to sampling effect or that the popularity of the domestic lineage of geese carrying this haplotype had decreased.

The genetic diversity was the highest during the High and Late Medieval Period according to the haplotype and nucleotide diversity estimates, probably due to high number of F-haplotypes. However, our sample sizes were not equal among the temporal groups, nor were the time periods the same length, all aspects that could bias our results. Although goose bones are reported to be more frequent in archaeological sites during the 13th–14th centuries [1,19], we did not detect signs of population growth in any of the temporal groups according to Tajima’s *D* and Fu’s *Fs.* The pairwise *Φ*_ST_ values showed an increase in genetic differentiation over time. The preceding time periods were genetically similar as the pairwise *Φ*_ST_ values were low and non-significant but statistically significant values were detected between the temporarily most separated groups, namely the amalgamated High and Late Medieval and the Present Period. Overall, the genetic differentiation was gradual and detected only over longer time spans. 

The presence of taiga bean geese among the subfossil samples was unexpected as all the bones were classified as domestic or domestic/wild greylag goose based on the morphology and no other species were selected for this study. The taiga bean goose bones can either be from wild hunted individuals misidentified as domestic goose, from domesticated bean geese or from domestic geese hybridized with bean geese. The similarly sized goose species are difficult to identify by species as has been noted also in a previous study [14], especially from fragmented or poorly preserved material. This most probably explains the presence of taiga bean geese in our sample. Bean geese migrate through the Middle Volga Region during the spring and autumn migrations [93] and have done so in the past as well [94,95,96,97,98,99] (see Text S1). It is possible, but very unlikely, that the bean goose was domesticated at least to a certain degree which made the bones morphologically more similar to the typical domestic goose. No evidence exists for this, as only the greater white-fronted goose (*Anser albifrons*) and the Canada goose (*Branta canadensis*) have been domesticated on experimental basis in ancient or historical times [2,7]. The hybridization of the domestic goose and the bean goose is plausible since hybrids between these species have been observed at least in captivity [100]. Again, only wild greater white-fronted geese have been known to be bred with domestic geese, to create a breed called Pskov bald (see Text S1). All the taiga bean goose fossils are from the earliest time periods, the Early and High Medieval Period (4th to 13th centuries), which could indicate that wildfowling was more common during this era or that the two species are easier to discriminate from the more recent samples. 

Our study provides the basis for aDNA analysis of domestic goose also over larger geographic areas and broader time frames. The analysis of ancient samples from the area of possible goose domestication including Egypt, ancient Mesopotamia, Turkey and Greece and nearer the time when domestication would have occurred would greatly help pinpoint the timing and location of the domestication event(s). However, possible problems may arise with DNA preservation in hot and humid conditions of the Mediterranean region [101]. An additional benefit of further sampling of both domestic and wild geese is the information it would provide on changes in goose husbandry versus wildfowling and on the economic activities of people.

## 5. Conclusions

We traced over a thousand years of evolutionary history of the European domestic goose through the Medieval and the Post-Medieval Periods in Russian archaeological sites, especially in the Middle Volga Region. We identified three genetic lineages among the samples: D-haplogroup, F-haplogroup, and the taiga bean goose. We found that geese of the typical domestic goose haplogroup D were present at least from the High Medieval Period (11th century CE) onward. However, the origin of the geese carrying the F-haplotypes is less certain, as the haplotypes found are not present among modern domestic geese. Surprisingly, we also found bean goose haplotypes, even from goose bones classified as “domestic.”

## Figures and Tables

**Table 1:** Population summary statistics for the concatenated hypervariable part of the mitochondrial DNA (mtDNA) control region (204 bp) of the domestic goose comparing the Present Period, the Post-Medieval Period (16th–18th centuries) and the High and Late Medieval Periods (11th–15th centuries). Sample size (*n*), number of haplotypes (*H*), haplotype (*h*), and nucleotide diversity (*π*) with standard deviations (SD) and Tajima’s *D* and Fu’s *Fs* for testing population expansion. Statistically significant values of comparisons with the Present Period are indicated.

Period	*n*	*H*	*h* (SD)	*π* (SD)	*D*	*Fs*
Present	102	7	0.584 (0.030)	0.0056 (0.0012)	−1.207	−0.753
Post-Medieval	22	3	0.541 * (0.068)	0.0056 (0.0028)	−1.573	1.652
High and Late Medieval	16	3	0.658 ** (0.075)	0.0134 † (0.0042)	0.482	3.924

* *t* = 2.89, d.f. = 23, *p <* 0.01; ** *t =* 3.90, d.f. = 16, *p* < 0.001; † *t =* 7.38, d.f. = 15, *p* < 0.001.

**Table 2:** Pairwise *Φ*_ST_ values for the concatenated hypervariable part of the mitochondrial DNA (mtDNA) control region (204 bp) of the domestic goose between the Present Period, the Post-Medieval Period (16th–18th centuries) and the High and Late Medieval Periods (11th–15th centuries). Statistically significant values (*p* < 0.05) after Bonferroni correction indicated with an asterisk.

Time Period	Present (*n* = 102)	Post-Medieval (*n* = 22)
Post-Medieval (*n* = 22)	0.026	
High and Late Medieval (*n* = 16)	0.105 *	0.064

## Supplementary Materials

The following are available online at http://www.mdpi.com/2073-4425/9/7/367/s1, Table S1. List of the successfully analyzed subfossil goose samples from Russia; Table S2. Variable nucleotide sites; Text S1. Archaeological and historical context of the samples.
