# Supplementary material for: Over a Thousand Years of Evolutionary History of Domestic Geese from Russian Archaeological Sites, Analysed Using Ancient DNA
Source: Genes (Basel). 2018 Jul 20;9(7):367. doi: 10.3390/genes9070367 (PMC6070935; doi:10.3390/genes9070367)
Supplement: Supplementary file 1 [file genes-09-00367-s001.pdf]

Supplementary information for:

Over a thousand years of evolutionary history of domestic geese from Russian archaeological sites, analysed using ancient DNA

Johanna Honka, Matti T. Heino, Laura Kvist, Igor V. Askeyev, Dilyara N. Shaymuratova, Oleg V. Askeyev, Arthur O. Askeyev, Marja E. Heikkinen, Jeremy B. Searle and Jouni Aspi

Table S1. List of the successfully sequenced subfossil goose samples (*n* = 51) from Russia with their haplotype and species inferred from the concatenated hypervariable part of the mtDNA (mitochondrial DNA) control region sequences (204 bp). The haplotype names follow the nomenclature in [1] for domestic and greylag goose (*Anser anser*) and [2] for taiga bean goose (*A. fabalis fabalis*). Forward slashes between haplotype names denote haplotypes that differ over the whole control region sequence (1249 bp [1]) but cannot be distinguished in the 204 bp sequence analysed here. In addition, the archaeological information is listed with the species inferred from bone morphology, the studied skeletal element, the sample dating by the archaeological context, the assigned temporal period of the samples, the excavation site and the location of site.

Sample name	Haplo-type	Species based on mtDNA	Species based on bone morphology	Studied bone fragment	Archaeological dating	Time period	Archaeological site	Location of site
JH24	Fa5	Taiga bean goose	Domestic goose	ulna/humerus	4th -8th centuries CE	Early Medieval	Tetyushskoe II hillfort	Tatarstan Republic
JH25	Fa5	Taiga bean goose	Domestic goose	humerus	4th -8th centuries CE	Early Medieval	Tetyushskoe II hillfort	Tatarstan Republic
JH1	F11	Domestic goose/ Wild greylag goose	Domestic goose	tibiotarsus	4th -8th centuries CE	Early Medieval	Tetyushskoe II hillfort	Tatarstan Republic
JH23	F11	Domestic goose/ Wild greylag goose	Domestic goose/ Wild greylag goose	furcula	5th -7th centuries CE	Early Medieval	Imenkov hillfort	Tatarstan Republic
JH39	FAB1	Taiga bean goose	Domestic goose	tibiotarsus	9th - 10th centuries CE	Early Medieval	Staraya Ladoga	Leningrad Region
JH40	Fa3	Taiga bean goose	Domestic goose	femur	9th - 10th centuries CE	Early Medieval	Staraya Ladoga	Leningrad Region
JH37	F6	Domestic goose/ Wild greylag goose	Domestic goose/ Wild greylag goose	humerus	11th -12th centuries CE	High Medieval	Ostolopovskoe settlement	Tatarstan Republic
JH38	D3/D7	Domestic goose	Domestic goose/ Wild greylag goose	tarsometatarsus	11th -12th centuries CE	High Medieval	Ostolopovskoe settlement	Tatarstan Republic

JH29	Fa6	Taiga bean goose	Domestic goose/ Wild greylag goose	femur	11th -13th centuries CE	High Medieval	Bilyarsk (Defensive moat location- peripherals of settlement)	Tatarstan Republic
JH30	D3/D7	Domestic goose	Domestic goose	humerus	12th -13th centuries CE	High Medieval	Bilyarsk (Palace lord location - downtown)	Tatarstan Republic
JH31	D3/D7	Domestic goose	Domestic goose	tibiotarsus	12th -13th centuries CE	High Medieval	Bilyarsk (Palace lord location - downtown)	Tatarstan Republic
JH56	D4/D5	Domestic goose	Domestic goose/ Wild greylag goose	ulna	12th -13th centuries CE	High Medieval	Elabuga hillfort	Tatarstan Republic
JH28	D4/D5	Domestic goose	Domestic goose	sternum	13th -14th centuries CE	High Medieval	Bagaevskoe settlement	Saratov Region
JH47	D3/D7	Domestic goose	Domestic goose/ Wild greylag goose	humerus	13th -14th centuries CE	High Medieval	Nizhny Novgorod Kremlin	Nizhny Novgorod Region
JH48	D3/D7	Domestic goose	Domestic goose/ Wild greylag goose	tibiotarsus	13th -14th centuries CE	High Medieval	Nizhny Novgorod Kremlin	Nizhny Novgorod Region
JH49	D4/D5	Domestic goose	Domestic goose/ Wild greylag goose	tarsometatarsus	13th -14th centuries CE	High Medieval	Nizhny Novgorod Kremlin	Nizhny Novgorod Region
JH50	D4/D5	Domestic goose	Domestic goose/ Wild greylag goose	tibiotarsus	13th -14th centuries CE	High Medieval	Bulgar	Tatarstan Republic
JH51	F6	Domestic goose/ Wild greylag goose	Domestic goose/ Wild greylag goose	tibiotarsus	13th -14th centuries CE	High Medieval	Bulgar	Tatarstan Republic
JH52	Fa3	Taiga bean goose	Domestic goose/ Wild greylag goose	ulna	13th -14th centuries CE	High Medieval	Bulgar	Tatarstan Republic
JH53	D4/D5	Domestic goose	Domestic goose /Wild greylag goose	tarsometatarsus	13th -14th centuries CE	High Medieval	Bulgar	Tatarstan Republic
JH32	D3/D7	Domestic goose	Domestic goose	humerus	15th century CE	Late Medieval	Toretskoe settlement	Tatarstan Republic
JH33	D3/D7	Domestic goose	Domestic goose	humerus	15th century CE	Late Medieval	Toretskoe settlement	Tatarstan Republic
JH34	D3/D7	Domestic goose	Domestic goose	humerus	15th century CE	Late Medieval	Toretskoe settlement	Tatarstan Republic
JH35	F6	Domestic goose/ Wild greylag goose	Domestic goose	humerus	15th century CE	Late Medieval	Toretskoe settlement	Tatarstan Republic

JH16	D4/D5	Domestic goose	Domestic goose	radius	16th -17th centuries CE	Early Post-Medieval	Kazan city (territory of Kazan University)	Tatarstan Republic
JH17	D4/D5	Domestic goose	Domestic goose	humerus	16th -17th centuries CE	Early Post-Medieval	Kazan city (territory of Kazan University)	Tatarstan Republic
JH18	D4/D5	Domestic goose	Domestic goose	humerus	16th -17th centuries CE	Early Post-Medieval	Kazan city (territory of Kazan University)	Tatarstan Republic
JH2	D4/D5	Domestic goose	Domestic goose	humerus	16th -17th centuries CE	Early Post-Medieval	Kazan city (territory of Kazan University)	Tatarstan Republic
JH3	D4/D5	Domestic goose	Domestic goose	humerus	16th -17th centuries CE	Early Post-Medieval	Kazan city (territory of Kazan University)	Tatarstan Republic
JH4	D4/D5	Domestic goose	Domestic goose	humerus	16th -17th centuries CE	Early Post-Medieval	Kazan city (territory of Kazan University)	Tatarstan Republic
JH5	D4/D5	Domestic goose	Domestic goose	humerus	16th -17th centuries CE	Early Post-Medieval	Kazan city (territory of Kazan University)	Tatarstan Republic
JH6	D3/D7	Domestic goose	Domestic goose	coracoideum	16th -17th centuries CE	Early Post-Medieval	Kazan city (territory of Kazan University)	Tatarstan Republic
JH7	D4/D5	Domestic goose (possible duplicate of the humerus in this site)	Domestic goose	coracoideum	16th -17th centuries CE	Early Post-Medieval	Kazan city (territory of Kazan University)	Tatarstan Republic
JH8	D3/D7	Domestic goose	Domestic goose	coracoideum	16th -17th centuries CE	Early Post-Medieval	Kazan city (territory of Kazan University)	Tatarstan Republic
JH10	D3/D7	Domestic goose	Domestic goose	femur	16th -17th centuries CE	Early Post-Medieval	Kazan city (territory of Kazan University)	Tatarstan Republic
JH11	D4/D5	Domestic goose (possible duplicate of the humerus in this site)	Domestic goose	femur	16th -17th centuries CE	Early Post-Medieval	Kazan city (territory of Kazan University)	Tatarstan Republic
JH12	D4/D5	Domestic goose (possible duplicate of the humerus in this site)	Domestic goose	tibiotarsus	16th -17th centuries CE	Early Post-Medieval	Kazan city (territory of Kazan University)	Tatarstan Republic

JH13	D4/D5	Domestic goose (possible duplicate of the humerus in this site)	Domestic goose	tarsometatarsus	16th -17th centuries CE	Early Post-Medieval	Kazan city (territory of Kazan University)	Tatarstan Republic
JH14	D4/D5	Domestic goose (possible duplicate of the humerus in this site)	Domestic goose	carpometacarpus	16th -17th centuries CE	Early Post-Medieval	Kazan city (territory of Kazan University)	Tatarstan Republic
JH61	D3/D7	Domestic goose	Domestic goose	femur	16th -18th centuries CE	Early Post-Medieval	Cheboksary city	Chuvash Republic
JH62	D3/D7	Domestic goose	Domestic goose	humerus	16th -18th centuries CE	Early Post-Medieval	Cheboksary city	Chuvash Republic
JH64	D3/D7	Domestic goose	Domestic goose	tibiotarsus	16th -18th centuries CE	Early Post-Medieval	Cheboksary city	Chuvash Republic
JH45	D4/D5	Domestic goose	Domestic goose/ Wild greylag goose	tibiotarsus	first half of 16th century CE	Early Post-Medieval	Pskov city (New Torg location)	Pskov Region
JH46	D4/D5	Domestic goose	Domestic goose/ Wild greylag goose	tibiotarsus	first half of 16th century CE	Early Post-Medieval	Pskov city (New Torg location)	Pskov Region
JH54	D3/D7	Domestic goose	Domestic goose	humerus	17th – 18th centuries CE	Early Post-Medieval	Elabuga hillfort	Tatarstan Republic
JH55	D3/D7	Domestic goose	Domestic goose	tibiotarsus	17th – 18th centuries CE	Early Post-Medieval	Elabuga hillfort	Tatarstan Republic
JH65	D4/D5	Domestic goose	Domestic goose	humerus	17th century CE	Early Post-Medieval	Sviyazhsk	Tatarstan Republic
JH66	D4/D5	Domestic goose	Domestic goose	tibiotarsus	17th century CE	Early Post-Medieval	Sviyazhsk	Tatarstan Republic
JH58	F6	Domestic goose/ Wild greylag goose	Domestic goose	furcula	18th century CE	Late Post-Medieval	Kazan Kremlin	Tatarstan Republic
JH59	D4/D5	Domestic goose	Domestic goose	humerus	18th century CE	Late Post-Medieval	Kazan Kremlin	Tatarstan Republic
JH60	D4/D5	Domestic goose	Domestic goose	humerus	18th century CE	Late Post-Medieval	Kazan Kremlin	Tatarstan Republic

Table S2. Variable nucleotide sites in the concatenated 204 base pairs of the mitochondrial control region haplotypes discovered among ancient domestic goose samples from Russian archaeological sites. Haplotypes FAB1, Fa3, Fa5 and Fa6 belong to another species, the taiga bean goose (*Anser fabalis fabalis*), but were discovered among the morphologically identified domestic/wild greylag goose subfossil bones.

	**Position**																													
**Haplotype**	2	5	13	14	19	25	26	35	38	41	44	71	86	89	91	92	97	102	103	109	113	118	121	135	142	155	168	169	172	178
D4/D5	T	A	G	C	C	G	A	C	A	G	A	C	A	T	T	A	A	C	T	C	T	C	A	G	T	T	C	T	C	T
D3/D7	.	.	.	.	T	.	.	.	.	.	.	.	.	.	.	.	.	.	.	.	.	.	.	.	.	.	.	.	.	.
F6	.	.	.	T	T	.	.	.	G	A	.	.	G	C	.	.	.	.	.	.	.	.	.	.	.	C	.	.	.	C
F11	.	.	.	T	T	.	.	.	G	A	.	.	G	C	.	.	.	.	.	.	.	.	.	.	.	C	.	C	.	.
FAB1	A	G	A	T	T	A	G	T	.	.	G	T	.	.	C	G	G	T	C	T	C	T	C	A	C	.	T	C	T	C
Fa3	A	G	A	T	T	A	G	T	.	.	G	T	.	.	C	G	G	T	C	T	C	T	C	A	C	.	T	.	T	C
Fa5	A	G	A	T	T	A	G	T	.	.	G	T	.	.	C	.	G	T	C	T	C	T	C	A	C	.	T	C	T	C
Fa6	A	.	A	T	T	A	G	T	.	.	G	T	.	.	C	G	G	T	C	T	C	T	C	A	C	.	T	.	T	C

Text S1. Archaeological and historical context of the samples

1. Archaeological record

Based on bird bones from archaeological sites from the European part of Russia and Ukraine, it can be concluded that the first authentic appearance and existence of domestic geese in this area occurred in the 6th century BCE - 4th century CE in the northern Black Sea region [3-6]. In this area, ancient Greek city-colonies (Olbia, Phanagoria, Tanais, etc.) and states such as the Bosporan kingdom existed [7]. The archaeological remains of domestic goose can be found in many Medieval settlements, both urban and rural, in Ukraine, Belarus and the European part of Russia, with the exception of the northern regions [4-6,8-13]. In western Siberia, the domestic goose appeared at the end of the 16th century and at the beginning of the 17th century as a result of the Russian colonisation of Siberia and the establishment of large trade settlements [14-15].

2. Historical context

2.1. Local goose breeds in the vicinity of the Tatarstan Republic

In some remote villages inhabited by Udmurts and Maris up to the 1970s, the breeding of strictly local geese took place, which had the appearance and behavioural characteristics of greylag geese *(Anser anser)* [16]. These geese flew very well, had very good parenting skills, laid eggs and incubated them without human help, the colour of feathers and the body size was the same as that of wild geese and these geese also made flights far from home [16]. But over the past 50 years, apparently, the practice of breeding these geese has not survived. In the Urals, at the end of the 17th century, the Shadrin breed was established on the basis of interbreeding of local wild geese and a local breed of domestic goose [17]. This breed did not experience the inflow of genes from other breeds of geese. Only small numbers of this breed currently survive [17].

A very interesting breed of geese was created in the 19th century in the Pskov Region, called the Pskov bald goose [17]. It was obtained as a result of crossing local domestic geese with wild greater white-fronted geese (*A. albifrons*). The average goose has a horizontal body, red-orange legs and bill, a blue or light-grey plumage with a white spot on the forehead [17].

2.2. Practices of egg and gosling collection

Extensive data exist in the historical and ornithological literature about collecting goose eggs and catching goslings for their further rearing in captivity in Ukraine, Russia and Kazakhstan. In the middle of the 16th century, Miehalonis Litnani [18] reported on the practice of collecting wild goose eggs to obtain goslings for captive rearing in the Dnieper Region of Ukraine. P.I. Rychkov [19] describes that in the middle of the 18th century, the inhabitants of the new fortresses in Orenburg Province caught goslings of the greylag goose, and raised them together with domestic geese. In the Urals, at the end of the 19th century and the first third of the 20th century, the Bashkirs practised collection of wild greylag goose eggs and putting them under domestic geese in order to produce goslings [20]. Rearing of goslings of grey geese caught from the wild was also practised in the first third of the 20th century in several regions of Kazakhstan [21,22]. The most widespread captive breeding of the wild greylag geese from eggs and catching of small goslings took place in the south of western Siberia, particularly in the Barabinsk Area (steppe) [22,23]. Quite recently, in the 1950s and 1970s, in the north of the Arkhangelsk Region and in the west of the Komi Republic, the local people practised catching goslings of the taiga bean goose (*A. fabalis*) for captive breeding [24,25]. Ornithologists and naturalists of the 19th and early 20th centuries noted that the locals of the Baikal and Amur Regions caught goslings of swan goose (*A. cygnoid*) for captivity, in which they were quickly tamed and kept until autumn [26,27].

2.3. Bean geese in the Tatarstan Republic

According to ornithological studies in the 19th and 20th centuries in the Middle Volga Region, the bean goose was a numerous migrating goose [28-33]. The numbers of various bean goose taxa varied considerably

between years in the Middle Volga Region at the end of the 19th century and the first third of the 20th century. So in the early 1890s, the tundra bean goose (*Melanonyx segetum = A. fabalis rossicus*) predominated to a considerable extent, with the main range spanning between Kazan and the Kama River and along the valley of this river. The taiga bean goose (*Melanonyx arvensis = A. fabalis fabalis*) occurred in small numbers with the main migration route following the Volga River [30]. By the late 1920s and early 1930s, in the same area, migratory flocks of the taiga bean goose (*M. arvensis = A. f. fabalis*) prevailed, and the numbers of the tundra bean goose (*M. segetum = A. f. rossicus*) were significantly smaller [31].

Monitoring of the spring migration (April-May) of the bean goose in the Tatarstan Republic (2016 - 2017) showed that the tundra bean goose (*A. f. rossicus*) predominated in the years 2016 (94.5 %) and 2017 (93.4 %) with taiga bean goose (*A. f. fabalis*) constituting 5.5 % and 6.6 %, respectively [34]. The taiga bean goose has a flyway along the valley of the Volga River to Kazan, and then along the valleys of the Ashit, Kazanka and Miesha Rivers and to the Vyatka River and along its valley northwards. The main flyway of the tundra bean goose goes from south of Kazan to the Kama River, and then along its valley eastwards and northeastwards. Fifteen bean geese obtained during the spring hunt in 2015 - 2017 from different parts of the Tatarstan Republic were also examined and morphologically identified. Of these, tundra bean goose comprised 14 specimens (93.3 %) and one specimen (6.7 %) was the typical taiga bean goose. Thus, both the taiga and the tundra bean goose migrate across the Middle Volga Region and have done so in the past.

References

1. Heikkinen, M.E.; Ruokonen, M.; Alexander, M.; Aspi, J.; Pyhäjärvi, T.; Searle, J.B. Relationship between wild greylag and European domestic geese based on mitochondrial DNA. *Anim. Genet.* **2015**, *46*, 485-497.
2. Honka, J.; Kvist, L.; Heikkinen, M.E.; Helle, P.; Searle, J.B.; Aspi, J. Determining the subspecies composition of bean goose harvests in Finland using genetic methods. *Eur. J. Wildl. Res.* **2017**, *63*, 19.
3. Voinstvensky, M.A. *Ornithofauna of Olbia. Archaeological Sites of Ukraine. No 7* (in Ukrainian); Institute of Archeology: Kiev, Ukraine, 1958.
4. Voinstvensky, M.A. *Fossil Avifauna of Ukraine* (in Russian); The Natural Environment and the Fauna of the Past: Kiev, Ukraine, 1967.
5. Burchak-Abramovich, N.I.; Tsalkin, V.I. To the knowledge of the avifauna of South Ukraine, Crimea and the Don Region (according to archaeological materials) (in Russian). *Bulletin of the Moscow Society of Naturalists (Biological series)* **1971**, *76*, 54-63.
6. Umanskaya, A.S. *Domestic Birds from Archaeological Sites of Ukraine* (in Russian); Natural Environment and Fauna of the Past: Kiev, Ukraine, 1972.
7. Koshelenko, G.A.; Kruglikova, I.T.; Dolgorukov, V.S., Eds. *Ancient States of the Northern Black Sea Littoral* (in Russian); Archeology of the USSR, Nauka: Moscow, Russia, 1984.
8. Burchak-Abramovich, N.I.; Tsalkin, V.I. Birds from archaeological excavations in the Moscow Kremlin (in Russian). *Bulletin of the Moscow Society of Naturalists (Biological series)* **1969**, *74*, 49-53.
9. Burchak-Abramovich, N.I.; Tsalkin, V.I. Materials for the study of European birds of RSFSR (according to archaeological sites) (in Russian). *Bulletin of the Moscow Society of Naturalists (Biological series)* **1972**, *77*, 51-59.
10. Bryuzgina (Umanskaya), A.S. Late Anthropogenic Birds from the Ukraine and Contiguous Territories (Primarily Based on Materials from Archeological Sites) (in Russian). Thesis for a Candidate of Biological Science, Institute of Zoology, Kiev, 1975.
11. Askeyev, I.V.; Galimova, D.N.; Askeyev, O.V. Birds of the Middle Volga Region during the V – XVIII centuries AD (according to archaeological excavations) (in Russian). *Volga River Region Archaeology (Zhurnal Povolzskaya Arkheologiya)* **2013**, *3*, 116-144.
12. Galimova, D.N.; Askeyev, I.V.; Askeyev, O.V. Bird remains from 5th - 17th century AD archaeological sites in the Middle Volga region of Russia. *Int. J. Osteoarchaeol.* **2014**, *24*, 347-357.
13. Gorobets, L.; Kovalchuk, O. Birds in the medieval culture and economy of the East Slavs in the 10-13th centuries AD. *Environ. Archeol.* **2017**, *22*, 147-165.
14. Nekrasov, A.E. *Bone Remains of Birds from the Holocene Locations of the Urals and Western Siberia* (in Russian); Quaternary paleozoology in the Urals: Yekaterinburg, Russia 2003.
15. Martynovich, N.V. Birds of "Gold-Fired" Mangazeya (in Russian). *Zoological Journal* **2013**, *92*, 1129-1135.

16. Askeyev, I.V.; Askeyev, O.V. Unpublished data.
17. Fisinin, V.I.; Zlochevskaya, K.V. Geese. In *Animal Genetic Resources of the USSR*; Dmitriev, N.G. and Ernst, L.K., Eds.; FAO: Rome, Italy, 1989, pp. 469-506.
18. Antonovich, V., Ed. *Memoirs Related to the History of Southern Russia, issue 1 (16th century)* (in Russian); Korchak-Novitsky: Kiev, Ukraine, 1890.
19. Rychkov, P.I. *Topography Orenburg, that is a Detailed Description of the Orenburg Province. Part 1* (in Russian); The Imperial Academy of Sciences: St. Petersburg, Russia, 1762.
20. Rudenko, S.I. *The Bashkirs: Historical-Ethnographic Essays* (in Russian); Academy of Sciences of the USSR: Moscow, Russia, 1955.
21. Spangenberg, E.P.; Feigin, G.A. Hunting birds of Kyzylorda District of Syrdarya Region (in Russian). *Proceedings of the Forest Experimental Business* **1930,** *VII*, 157-192.
22. Tugarinov, A.J. *Anseriformes* (in Russian); Fauna of USSR Birds, Vol. 1 (4). Edition De L'Academie Des Sciences De l'URSS: Moscow-Leningrad, Russia, 1941.
23. Zverev, M.D. *Predatory Ways of Catching Commercial Birds in the Barabinsk Steppe* (in Russian); News of the Siberian Regional Scientific Hunting and Animal Production Station no. 1: Novosibirsk, Russia, 1930.
24. Spangenberg, E.P. *Goose Country. Birds, Hares, Foxes and Others ... Stories of the Naturalist* (in Russian); Children's Literature Publishing House: Moscow, Russia, 1973.
25. Krivushev, A.V.; Vlasov, M.A.; Matev, V.E. Bean goose in the Udorsk Region of the Komi Republic (in Russian). *Casarca* **2000**, *6*, 87.
26. Middendorf, A. *Travel to the North and East of Siberia 2, 5 Siberian Fauna* (in Russian); St. Petersburg, Russia, 1869.
27. Shulpin, L.M. *Commercial, Hunting and Predatory Birds of Primorye* (in Russian); Far Eastern Branch of the Academy of Sciences of the USSR: Vladivostok, Russia, 1936.
28. Eversmann, E. *Natural History of Birds of Orenburg Territory* (in Russian); Kazan University Press: Kazan, Russia, 1866.
29. Bogdanov, M.N. Birds and mammals in the blacksoil zone of the Volga Region and in the valleys of the Middle and Lower Volga river (in Russian). *Proceedings Kazan Naturalist of the Society* **1871**, *1*, 3–226.
30. Ruzsky, M.D. The results of investigations of birds in the Kazan province (in Russian). *Proceedings Kazan Naturalist of the Society* **1893**, *25*, 1-394.
31. Bashkirov, I.S.; Grigoryev, N.D. *Essay on the Hunting of Tataria* (in Russian). Works of the Volga-Kama Regional Commercial - Biological Station v.1: Kazan, Russia 1931.
32. Artemiev, J.T.; Popov, V.A. *Order of Anseriformes. The Birds of the Volga-Kama Region: Non-Passeriformes* (in Russian). Popov, V.A., Ed.; Nauka: Moscow, Russia, 1977.
33. Askeyev, I.V.; Askeyev, O.V. *Birdfauna of Tatarstan Republic* (in Russian); Akademia Nauk Tatarstana: Kazan, Russia, 1999.
34. Askeyev, I.V.; Askeyev, O.V.; Askeyev, A.O. Unpublished data.
